# Artificial intelligence-assisted analysis of endoscopic retrograde cholangiopancreatography image for identifying ampulla and difficulty of selective cannulation

**DOI:** 10.1038/s41598-021-87737-3

**Published:** 2021-04-16

**Authors:** Taesung Kim, Jinhee Kim, Hyuk Soon Choi, Eun Sun Kim, Bora Keum, Yoon Tae Jeen, Hong Sik Lee, Hoon Jai Chun, Sung Yong Han, Dong Uk Kim, Soonwook Kwon, Jaegul Choo, Jae Min Lee

**Affiliations:** 1 Graduate School of Artificial Intelligence, KAIST, Daehak-ro 291, Yuseong-gu, Daejeon, 34141 Korea; 2 Division of Gastroenterology and Hepatology, Department of Internal Medicine, Korea University College of Medicine, Korea University Medical Center, Goryeodae-ro 73, Seongbuk-gu, Seoul, 02841 Korea; 3 Department of Internal Medicine, Pusan National University College of Medicine, Pusan, Korea; 4 Department of Anatomy, Catholic University of Daegu, Daegu, Korea

**Keywords:** Endoscopy, Computer science

## Abstract

The advancement of artificial intelligence (AI) has facilitated its application in medical fields. However, there has been little research for AI-assisted endoscopy, despite the clinical significance of the efficiency and safety of cannulation in the endoscopic retrograde cholangiopancreatography (ERCP). In this study, we aim to assist endoscopists performing ERCP through automatic detection of the ampulla and the identification of cannulation difficulty. We developed a novel AI-assisted system based on convolutional neural networks that predict the location of the ampulla and the difficulty of cannulation to the ampulla. ERCP data of 531 and 451 patients were utilized in the evaluation of our model for each task. Our model detected the ampulla with mean intersection-over-union 64.1%, precision 76.2%, recall 78.4%, and centroid distance 0.021. In classifying the cannulation difficulty, it achieved the recall of 71.9% for the class of easy cases and that of 61.1% for that of difficult cases. Remarkably, our model accurately detected AOV with varying morphological shape, size, and texture on par with the level of a human expert and showed promising results for recognizing cannulation difficulty. It demonstrated its potential to improve the quality of ERCP by assisting endoscopists.

## Introduction

Endoscopic retrograde cholangiopancreatography (ERCP) is an important procedure for patients with biliary and pancreatic disorders. The steps of the ERCP procedure are: moving the endoscope to the second duodenal portion, finding the ampulla, and cannulating the common bile duct (CBD) using a sphincterotome or catheter^1^. Among these steps, deep selective cannulation through CBD is the most important for not only the success of the procedure, but also the prevention of adverse events^2^. However, because there exists anatomic variations in the ampulla of Vater (AOV), such as diverticulum, it is often difficult to accurately identify the location of the ampulla in the duodenum, especially for inexperienced endoscopists. It results in a nontrivial delay of cannulation in ERCP, potentially degrading the quality of the procedures.

According to clinical guidelines, the cannulation in ERCP is usually considered delayed when any one of the following conditions is met: the cannulation time exceeds 5 min, repeated cannulation more than five times, or occurrence of unintended pancreatic duct manipulations^3^. Even when a well-experienced endoscopist performs ERCP in referral centers, the rate of cannulation failure could be as high as 20%^4^. Repetitive attempts of cannulation and inadvertent injection of contrast dye into the pancreatic duct are related to a high incidence of post-ERCP adverse events. Although it is common to perform an ERCP procedure under the supervision of a well-experienced endoscopist, this support is not often available in practice. Moreover, successful cannulation depends not only on the experience of the endoscopist but also on various anatomical factors and underlying disease among patients^5^.

Recently, novel artificial intelligence (AI) technology has made remarkable progress. Deep learning-based computer vision technologies surpassed even human performance in major image analysis tasks^6–10^. With this progress in AI, significant attention was paid to its potential application in the medical field^11–17^. For example, Takiyama et al.^16^ showed that convolutional neural networks (CNNs) could distinguish anatomical locations in esophagogastroduodenoscopy images. Hirasawa et al.^17^ endeavored to detect and localize early gastric cancer in endoscopic images and achieved high-quality results. Kim et al.^25^ developed a CNN-based automatic software system that predicted bone age using left-hand wrist radiography images and reduced the reading times of radiologists by 29% on average in daily clinical practice. Furthermore, Buetti-Dinh et al.^26^ proposed a CNN-based model that substantially outperformed human experts in predicting the species composition of bacterial biofilms. These results show that CNN-based models effectively capture various anatomical factors of a patient from the medical images, resulting in an accurate diagnosis. However, despite its effectiveness and the importance of endoscopic procedures, there have been few studies for assisting the ERCP procedure in pancreaticobiliary endoscopy with AI. Therefore, we formulated our own dataset and investigated the potential of CNN-based models to perform the aforementioned tasks and provide insights into distinguishing the anatomical features in endoscopic images.

In this study, we aim to propose an AI-assisted ERCP procedure to not only accurately detect the location of AOV in endoscopic images, but also estimate the difficulty of cannulation in advance.

## Materials and methods

### Study design, patients, and endoscopic procedure

This study was conducted at two tertiary medical centers via the collaboration between the Department of Internal Medicine and the Graduate School of AI in Korea. The endoscopic data were collected between January 2016 and December 2018 from the patients who had undergone ERCP at two tertiary medical centers in Korea. The patients who had underwent the ERCP procedure and had no history of the previous sphincterotomy were included. All the ERCP procedures were performed by four well-experienced endoscopists (two endoscopists per center). Among them, two endoscopists had more than 5 years of experience in ERCP, one had more than 10-years’ experience, and one had more than 20-years’ experience. All subjects were included after providing informed consent on collecting ERCP data before procedures. This study was conducted in accordance with the Declaration of Helsinki and approved by ‘InstitutionalReview Board of Korea University Anam Hospital’ (IRB #:2019AN0253). According to the recommendation from 'Human Research Ethics Committee of Korea University Anam Hospital', all acquired data was saved and analyzed in the assigned secure device. All personal information was de-identified and managed by assigned members only.

### Device and definition

ERCP was performed using a duodenoscope (JF 240 or TJF-260 V; Olympus Medical Systems Co. Ltd., Tokyo, Japan), and the endoscopic images were taken using video endoscopy systems (EVIS LUCERA CLV 260; Olympus Medical Systems Co. Ltd., Tokyo, Japan). The following endoscopic images were selected: (1) endoscopic images of the front on ampulla, (2) endoscopic images of the naïve ampulla before being touched by the endoscopic device, (3) endoscopic images taken after washing off bubbles and food materials from the duodenum.

The cannulation time was defined as the total time spent from approaching the ampulla to performing a successful deep cannulation into the CBD. Additionally, the data were obtained from the records written by the endoscopists regarding whether additional cannulation techniques were used or not. Additional cannulation techniques that were included were the double-guidewire technique, needle-knife fistulotomy, and changing the cannulation device after an initial trial. An instance of cannulation was considered a difficult case when the cannulation time exceeded 5 min, when additional techniques were used, or when the cannulation failed.

For the ampulla-detection task, each captured endoscopic image was annotated with a bounding box (bbox) that indicates the location of AOV. The bbox annotation comprises four real values, namely, the x and y coordinates of the upper-left point, and the width and height of the box. An endoscopist who had more than five years of experience in ERCP and conducted the procedure more than 2000 times performed the annotation.

### Detection of the location of AOV in the duodenum

This paper aims to assist endoscopists performing ERCP through two tasks, the automatic detection of the ampulla and the identification of cannulation difficulty. The former will be introduced in this section, and the latter will be explained in detail in the following section. Given the annotated location of AOV as bboxes, it is common to design a neural network to estimate them, i.e., to generate the real-valued output bboxes. However, in our model, instead of predicting bboxes that identify the exact range of the entire AOV, we estimate the probability of whether each image pixel belongs to an AOV. A strict bbox that divides AOV from non-AOV is not suitable for this task because the AOV is not strictly distinguished from the background. Rather, the AOV gradually blends into the background, similar to how a probability distribution continually ranges from 1 to 0. Predicting the exact range of bboxes that indicate AOV is a difficult task due to variations in the data (e.g. the boundary of AOV may be ambiguous, its morphology varies across patients). Such methods have been proven to be effective for datasets that similarly have ambiguous boundaries (Kim et al.^18^). In this sense, we made our model generate the output as a pixel-wise soft mask, which is a density map with the probability of whether each pixel belongs to an AOV, instead of a strict bbox.

**Figure 1:**
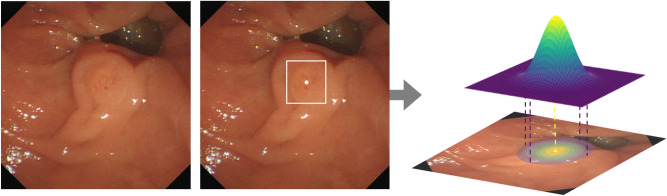
An example of an endoscopic image (left), its original bbox annotation (middle), transformed pixel-wise soft mask label (right). The centroid of the bbox is marked as a dot. For labels (middle and right), the image was overlapped to visualize the location of them on the image.

To this end, we transformed the bbox annotations into pixel-wise soft masks. Figure 1 illustrates a sample image with the original bbox annotation and the new soft mask label. First, regarding the possibility of pixels belonging to AOV, the centroid of the bbox should have the maximum probability while the probability should become smaller as the pixel is further away from the centroid. In addition, given that the shape of the ampulla is similar to a circle, not a rectangle, a bivariate normal distribution parametrized by the mean and covariance matrix was used to render masks. It is a bell-shaped curve, which has a maximum value at the mean and decreasing values with the speed determined by the covariance matrix. To formulate the new label, we set the mean as the centroid of the bbox annotation and the horizontal and the vertical variance as the half of the width and the height, respectively, of the box. As the width of the box becomes longer, the curve decreases more slowly along the corresponding axis.

In this manner, the new annotation is a pixel-wise soft mask whose values follow a bell-shaped probability distribution. While learning to minimize the difference between the outputs and ground truth (GT) labels using the binary cross-entropy loss, the network learns to assign the maximum probability at the centroid of the bbox and the shape of its outputs becomes close to a circle or an oval. During the evaluation, the output of the model is transformed back to a bbox form. The bbox includes the peak of the predicted mask and only those pixels with a probability larger than a threshold around the peak. We normalized the predicted mask to have a maximum value of one at peak and set the probability threshold as 0.6 to determine the boundary of the box.

For our neural network architecture, we adopted U-Net^19^ with a VGGNet-based^20^ encoder and decoder. VGGNet is a CNN model with stacked blocks of multiple $3 \times 3$ convolutional layers followed by a max pooling layer. U-Net consists of an encoder and a decoder with a residual connection for precise localization of objects within images. Also, we utilized transfer learning for faster learning and improved prediction accuracy, by fine-tuning our model pre-trained on the ImageNet dataset^21^.

### Classification of the ampulla-cannulation difficulty

After the location of AOV is detected, our next goal is to classify the endoscopic images of the duodenum according to the cannulation difficulty in ERCP. Depending on how the difficulty labels were grouped, we conducted the prediction in the following two ways: binary classification and four-class classification. First, we divided all the cases into two groups, namely, “easy case” or “difficult case” group. The “difficult case” group included the cases that had the cannulation time of over 5 min, cases requiring additional cannulation techniques, and failure of selective cannulation, except easy cases, as stated earlier. Furthermore, the groups were subdivided into four-class as follows: easy class, class whose cannulation time was over 5 min, class requiring additional cannulation techniques, and failure class.

Similar to the AOV detection task, CNN-based classification models were used and transfer learning was adopted. Specifically, modified versions of VGG19 with batch normalization^20^, ResNet50^22^, and DenseNet161^23^ were used. VGG19 is a VGGNet architecture with 19 layers, while batch normalization is a technique that keeps the distribution of activation values in a network constant. ResNet is a CNN model that allows residual mappings by adopting skip connections between the layers and effectively alleviates the gradient vanishing problem. DenseNet uses skip connections “densely” to maximize the advantage of skip connections. A single three-channel endoscopic image is used as an input to the model. While training, data were augmented every iteration by applying various transformations to endoscopic images, e.g., flipping, shearing, and rotating. We also used early stopping to avoid overfitting. All the networks in this study were implemented with the deep learning framework called PyTorch^24^.

### Outcome measures

When evaluating the model performance for the ampulla detection task, the following methods were used other than main outcome measures such as recall.

- Centroid distance is a relative coordinate error between centroids of the GT bbox and the estimated bbox. Its mathematical expression is as follows:
  1 $$centroid distance = \frac{1}{2}\left( {\frac{{\left| {x_{g} - x_{e} } \right|}}{W} + \frac{{\left| {y_{g} - y_{e} } \right|}}{H}} \right)$$
  where $\left( {x_{g} , y_{g} } \right)$, $\left( {x_{e} , y_{e} } \right)$, $W$, and $H$ denote coordinates of the GT bbox, those of the measured bbox, the width and height of the image, respectively.
- A success plot shows success rates for decreasing (or increasing) mean intersection-over-union (mIoU) (or centroid distance) thresholds. It is counted as a success if the IoU (or centroid distance) between the model prediction and the GT label for an image is bigger (or lower) than the threshold.
- Human performance was compared with our model. We randomly sampled 30 images from our test set of the first fold using the Python NumPy library and an expert endoscopist conducted the ampulla detection on the sampled data. The performance of our model was measured for the same images.

## Results

The baseline characteristics of the patients and the results of ERCP are listed in Table 1. A total of 531 patients were included in this study. Their mean age was 66.0 ± 15.2 years, and 303 (57%) patients were men. The median value of the cannulation time was 130.0 ± 305.5 s. The cannulation time was over 5 min in 69 patients, and there were 6 cases of cannulation failure. Additional techniques were used in 94 patients, and in all cases where additional techniques were used, the cannulation time exceeded 5 min. Finally, 169 cases (31.8%) were considered to have experienced cannulation difficulty. For the study of detecting AOV in the duodenum, the endoscopic images of 451 patients were selected for the task of annotating the AOV location in the endoscopic images. A total of 80 images were excluded because of poor image quality or ambiguous AOV location. All the 531 cases were used to estimate the cannulation difficulty.

**Table 1:** Baseline characteristics of the enrolled patients who underwent ERCP.

	Value
Patients, *n*	531
Age, mean ± SD (years)	66.0 ± 15.2
Male, *n* (%)	303 (57)
**ERCP findings, n (%)**	
Biliary gallstone disease	289 (54)
Malignant bile duct stricture	104 (20)
Cholangitis or papillitis only	72 (14)
Postoperative adverse event	19 (4)
Benign biliary stricture	15 (3)
Others	32 (6)
**Outcomes of ERCP**	
Success, *n* (%)	525 (99)
< 5 min	362 (68)
≥ 5 min	163 (31)
Failure, *n* (%)	6 (1)
Cannulation time, median ± SE (s)	130.0 ± 305.5

*ERCP* endoscopic retrograde cholangiopancreatography, *SD* standard deviation, *SE* standard error.

**Figure 2:**
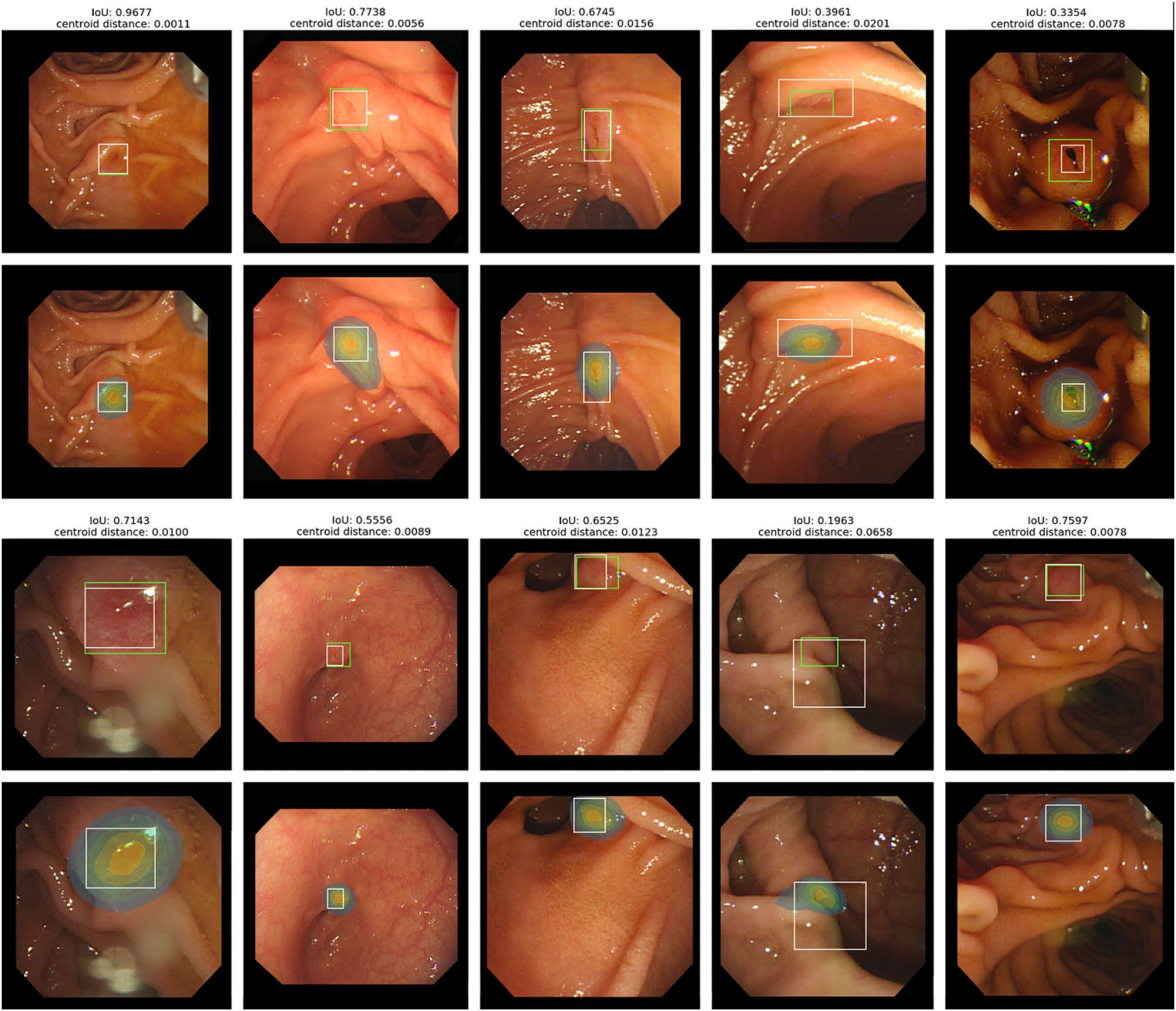
Examples of the model prediction and GT label. A model prediction is present in two different ways, the green bbox (upper) and heatmap visualization (lower). In both cases, the white bbox indicates the GT label. For each prediction, IoU and centroid distance are written above. Heatmap results show that the predicted masks from our model accurately match AOVs in size and shape, even ones with IoU around 30%.

**Figure 3:**
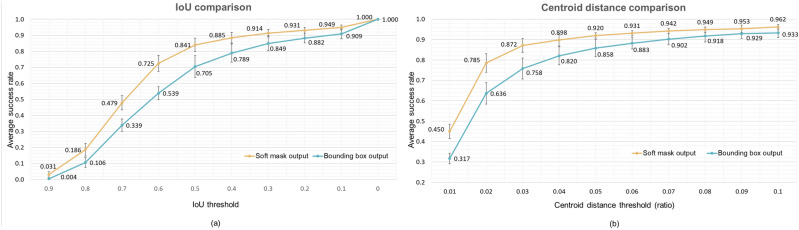
(**a**) The success plot with decreasing IoU threshold. The model that predicts soft masks is superior to the model that directly predicts bboxes. The former has a success rate of 91.4% for the threshold of 0.3, showing sufficient performance to assist the ERCP procedure. (**b**) The success plot with increasing centroid distance threshold. The model with soft mask output always performs better than the model with bbox output. Also, the former achieved a high success rate for the challenging threshold of 2%.

For all experiments, we performed fivefold cross-validation on our dataset and all results were reported as the average of the five folds with the standard deviation unless noted otherwise. In the ampulla location prediction task, the prediction results of our model were as follows: mIoU 0.641 ± 0.021, precision 0.762 ± 0.035, recall 0.784 ± 0.006, and centroid distance 0.021 ± 0.003. Examples of the model output and the GT label are shown in Fig. 2. The images with IoU ranges from 0.196 to 0.968 and centroid distance ranges from 0.001 to 0.066 are included. Figure 3 shows two success plots, one with IoU thresholds ranging from 0.0 to 0.9 and the other with increasing centroid-distance-ratio thresholds ranging from 0.01 to 0.1. As shown in Fig. 3a, the proposed method with soft mask output achieved the average success rate of 91.4 ± 2.3%, for 0.3 IoU threshold, compared to the model with bbox outputs with 84.9 ± 4.7%. Similarly, Fig. 3b shows the average success rate of the proposed model for having a lower centroid distance than 5% of the image resolution is 92.0 ± 1.28%, compared to 84.9 ± 4.7% of the bbox output model.

On the comparison between the proposed method and the human expert, our model scored mIoU 0.684 ± 0.185, precision 0.789 ± 0.215, recall 0.825 ± 0.207, and centroid distance 0.014 ± 0.016, while the human performance was mIoU 0.554 ± 0.199, precision 0.917 ± 0.106, recall 0.602 ± 0.245, and centroid distance 0.012 ± 0.010. When compared with the human expert instead of the GT label, our model achieved mIoU 0.514 ± 0.223, precision 0.570 ± 0.272, recall 0.869 ± 0.213, and centroid distance 0.008 ± 0.010.

For classifying the difficulty of cannulation, the performance results of the CNN-based models on the binary classification are shown in Table 2. Of all, ResNet achieved the best performance. We achieved the average recall of 0.719 ± 0.081 for the easy class and that of 0.611 ± 0.098 for the difficult class. Also, the F1-score was 0.757 ± 0.062 for the easy class and 0.553 ± 0.062 for the difficult class on average. As shown in Table 3, our best model scored a macro-average F1-score of 0.429 ± 0.062 and an accuracy of 0.667 ± 0.078 on the four-class classification task. The recall was 0.8039 for the easy class and 0.5638 for the class of cases requiring additional cannulation techniques on the four-class classification.

**Table 2:** Performance for binary classification of cannulation difficulty.

Model	VGG		ResNet		DenseNet	
	Easy	Difficult	Easy	Difficult	Easy	Difficult
Precision	0.787 ± 0.081	0.434 ± 0.083	**0.802** ± **0.047**	**0.507** ± **0.038**	0.772 ± 0.076	0.436 ± 0.047
Recall	0.607 ± 0.048	**0.656** ± **0.102**	**0.719** ± **0.081**	0.611 ± 0.098	0.631 ± 0.057	0.615 ± 0.045
F1 score	0.680 ± 0.016	0.511 ± 0.048	**0.757** ± **0.062**	**0.553** ± **0.062**	0.694 ± 0.061	0.507 ± 0.026
ACC	0.614 ± 0.024		**0.695** ± **0.042**		0.627 ± 0.037	
AUC	0.657 ± 0.061		**0.680** ± **0.021**		0.626 ± 0.034	

Bold fonts represent the best performance among the methods.

*AUC* area under the receiver operating characteristic curve, *ACC* accuracy.

**Table 3:** Performance for four-class classification.

Model	VGG	ResNet-macro F1	ResNet-ACC	DenseNet
Macro-average precision	0.360 ± 0.143	**0.444** ± **0.071**	0.340 ± 0.033	0.393 ± 0.110
Macro-average recall	0.342 ± 0.068	**0.445** ± **0.054**	0.328 ± 0.041	0.379 ± 0.067
Macro-average F1 score	0.304 ± 0.080	**0.429** ± **0.062**	0.364 ± 0.029	0.350 ± 0.095
Accuracy	0.691 ± 0.070	0.667 ± 0.078	**0.712** ± **0.056**	0.699 ± 0.064

Bold fonts represent the best performance among the methods.

*ResNet-macro F1* ResNet early stopped with macro-average F1-score, *ResNet-ACC* ResNet early stopped with accuracy.

## Discussion

Studies on AI in medicine have been widely conducted in recent years, and remarkable progress has been made. Notably, CNN-based models proved their potential in medical imaging applications and have been widely applied in the field of gastroenterology. For example, Constantinescu et al.^27^ adopted AI to detect polyps from endoscopic images and achieved 93.75% recall and 91.38% specificity, showing a similar diagnosis to a physician-led one that showed 94.79% recall and 93.68% specificity. Wu et al.^28^ proposed a CNN-based model that detected early gastric cancer and gastric locations better than endoscopists. Saito et al.^29^ obtained 98.6% accuracy in detecting protruding lesions from wireless capsule endoscopy images.

This study is the first one to develop an AI-based endoscopy support system for ERCP by comprehensively analyzing endoscopic images with clinical outcomes. The result has a clinical significance that the efficiency and safety of cannulation in the conventional ERCP might be improved via the support of the new AI technology. Although performing a perfect biliary cannulation has been a challenge for most endoscopists, an unsolved question still remains: what is the optimal cannulation technique for ERCP? We propose an AI-assisted system that detects the location of AOV and estimates the cannulation difficulty in advance while performing ERCP.

Our CNN-based models achieved competent performance in these tasks, especially in the ampulla detection task, showing robust performance on variations in data among patients, such as morphological shape, size, texture, location, and types of the diverticulum (Fig. 2). Moreover, our model even identifies the *shape* of the ampulla precisely, e.g., if the ampulla is vertically long or circular, so is the model output. These results are especially meaningful in that our model successfully detected both the location and morphological shape of AOV only with bbox annotations, not costly pixel-level annotations. Furthermore, performances of our model and the bbox output model are compared in Fig. 3. The former always shows better performance than the latter, proving the effectiveness of the soft mask.

Also, it is notable that even predictions with IoU between 0.3 and 0.4 adequately identified the location to practically assist the ERCP procedure. In this sense, we counted how many predictions achieved IoU bigger than 0.3. Remarkably, the average success rate was 91.4% (Fig. 3), showing that our model learned to detect ampulla in spite of the unclear boundary. Moreover, as shown in Fig. 3, the average success rate for the centroid distance threshold of 5% of the image resolution was 92.0%, where it can be easily seen through examples in Fig. 2 that 5% is a proper threshold to identify the performance. This result demonstrates that our model accurately detected the *location* of the AOV for most of the cases.

The comparison with the human expert results demonstrates that our model (mIoU 0.684, recall 0.825) achieved comparable performance with the human expert (mIoU 0.554, recall 0.602) in recognizing the range of AOV on average although the endoscopist (precision 0.917) was better at excluding unnecessary parts than our model (precision 0.789). Also, the centroid distance results show that its capability to pinpoint the location of AOV is on par with the level of a human expert.

Since the boundary of AOV is ambiguous, each annotator can draw different bounding boxes for the same AOV. In this sense, the difference between the human expert and the ground truth labels can be regarded as inter-annotator disagreement. Thus, we also measured the model performance regarding the human expert as a new GT. Although the mIoU and the precision are relatively small, the recall becomes even higher compared to when the original GT label is used (recall 0.825). This indicates that our model is not biased to the GT label and more similar to the expert than the GT label in terms of recall. Moreover, the centroid distance between our model and the human expert (0.008) is smaller than the distance between the GT label and the human expert (0.012). Even though our model never saw the expert's annotation while training, our model pinpoint AOV closer to the human expert than the GT label does. These results support that our model is generalizable.

In the task of binary classification of cannulation difficulty, our model showed high performance for estimating easy cases for selective cannulation with the average precision and recall of 0.802 and 0.719, respectively (Table 2). However, the selection of difficult cases remains to have a low recall of 0.611 on average. Therefore, more improvement would be required to use our model for clinical practice in ERCP.

On the other hand, there were some interesting and promising results in four-class classification. Although the estimation of a long cannulation time was low similarly to the binary classification, AI-assisted models showed a favorable performance in predicting the cases requiring additional technique (recall 0.564) even though only 17.70% of the data belong to this class. It shows the potential that endoscopic images solely can provide the information on whether additional techniques would be necessary while performing ERCP, without any repeated attempts and failures. Therefore, the use of the AI-assisted procedure had enough feasibility to get the additional information of ERCP.

**Figure 4:**
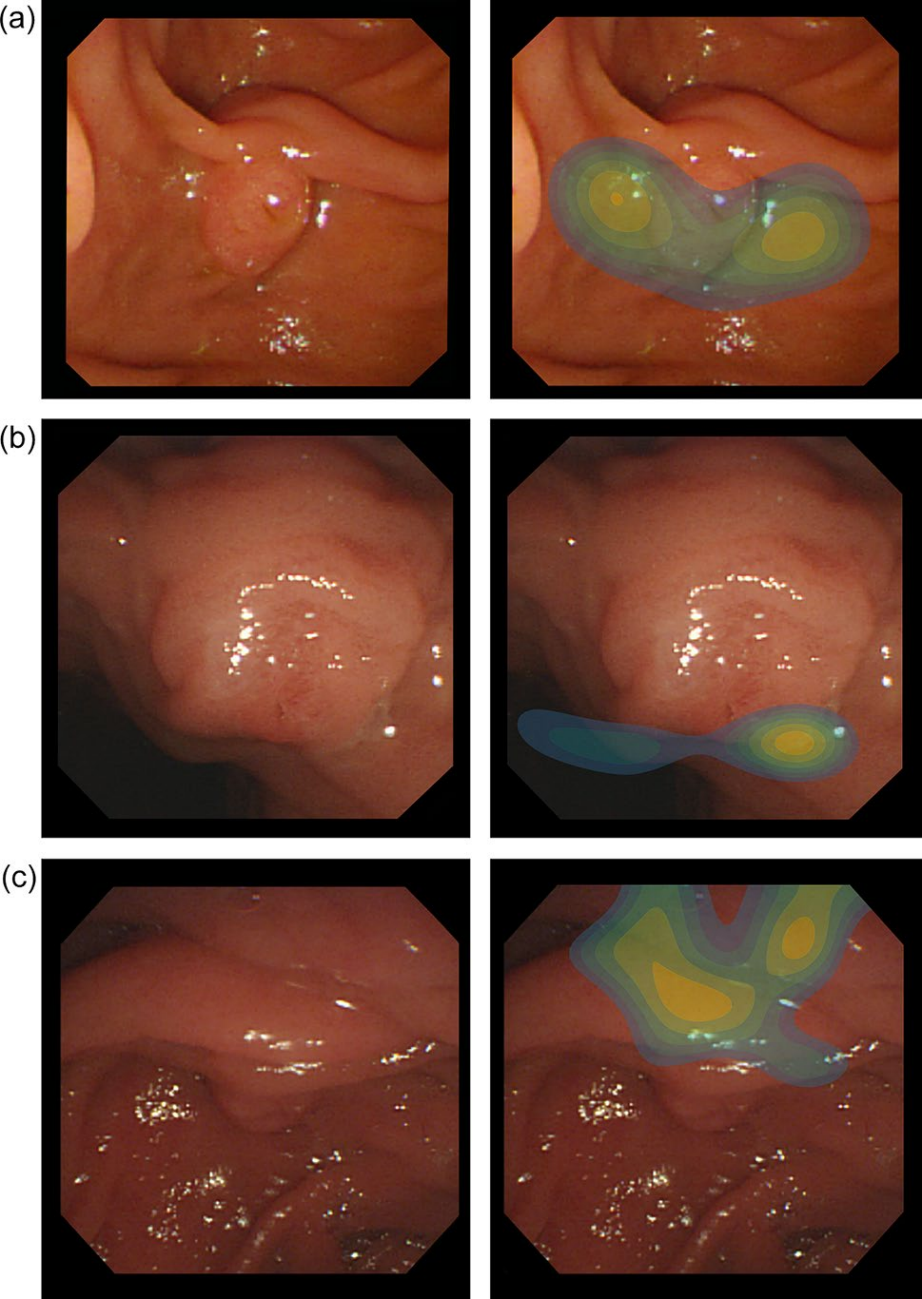
Grad-CAM results of the cannulation difficulty prediction model for accurately predicted examples. The heatmap visualizations are the outputs of Grad-CAM for the written GT label. They show where the model focused attention to estimate the cannulation difficulty. The lighter the color, the stronger is the attention required. The results show that the model sees distinct features of each label.

Additionally, in Fig. 4, we visualized class activation maps using gradient-weighted class activation mapping (Grad-CAM)^30^ to interpret the model behavior for correctly classified examples. It shows which visual features were meaningful to the model. The model focused on the surroundings of the ampulla for the easy cases and the bulging ampulla for cases of the more-than-five-min class. On the other hand, the upper crease of AOV was considered as an important feature for detecting cases requiring additional techniques. These results demonstrate that our model makes decisions according to learned features related to cannulation difficulties (possibly involving other visual features not mentioned) and that it can provide us further insights to explain the relationship between the various anatomical factors of patients and cannulation difficulties.

In future work, collecting additional information from radiologic data (e.g., computed tomography or magnetic resonance imaging) would improve the classification performance. For example, the inner structure of the ampulla is one factor that determines the cannulation difficulty but may not be easily captured from the endoscopic images. Also, data imbalance in difficulty classes needs to be addressed, which is one of the typical problems that degrade model performance in classification tasks^31–37^. Furthermore, sharing the learned knowledge between the cannulation difficulty prediction model and the ampulla detection model would lead to improvements in prediction accuracy for both tasks.

In conclusion, this study shows the potential of clinically applicable AI-based automatic ERCP procedures, even with a small number of data. The AI-assisted system showed high accuracy for finding the location of the ampulla at the duodenum in ERCP on par with the level of a human expert. It is expected to help make decisions in ambiguous situations during ERCP.

## Data Availability

The datasets generated during and/or analyzed during the current study are not publicly available due to patient’s privacy protection act.
